# MicroRNA-126 induces autophagy by altering cell metabolism in malignant mesothelioma

**DOI:** 10.18632/oncotarget.8916

**Published:** 2016-04-22

**Authors:** Marco Tomasetti, Federica Monaco, Nicola Manzella, Jakub Rohlena, Katerina Rohlenova, Sara Staffolani, Simona Gaetani, Veronica Ciarapica, Monica Amati, Massimo Bracci, Matteo Valentino, Jacob Goodwin, Maria Nguyen, Jaroslav Truksa, Margaryta Sobol, Pavel Hozak, Lan-Feng Dong, Lory Santarelli, Jiri Neuzil

**Affiliations:** ^1^ Department of Clinical and Molecular Science, Polytechnic University of Marche, 60020, Ancona, Italy; ^2^ Institute of Biotechnology, Czech Academy of Sciences, BIOCEV, Vestec-Prague West, 25242, Czech Republic; ^3^ School of Medical Science and Griffith Health Institute, Griffith University, Southport, Qld, 4222, Australia; ^4^ Institute of Molecular Genetics, Czech Academy of Sciences, Prague 4, 142 20, Czech Republic

**Keywords:** MIR126, malignant mesothelioma, autophagy, cell metabolism, tumor suppression

## Abstract

Autophagy favors both cell survival and cancer suppression, and increasing evidence reveals that microRNAs (*MIRs*) regulate autophagy. Previously we reported that *MIR126* is downregulated in malignant mesothelioma (MM). Therefore, we investigated the role of *MIR126* in the regulation of cell metabolism and autophagy in MM models. We report that *MIR126* induces autophagic flux in MM cells by downregulating insulin receptor substrate-1 (IRS1) and disrupting the IRS1 signaling pathway. This was specific to MM cells, and was not observed in non-malignant cells of mesothelial origin or in MM cells expressing *MIR126*-insensitive IRS1 transcript. The *MIR126* effect on autophagy in MM cells was recapitulated by IRS1 silencing, and antagonized by IRS1 overexpression or antisense *MIR126* treatment. The *MIR126*-induced loss of IRS1 suppressed glucose uptake, leading to energy deprivation and AMPK-dependent phosphorylation of ULK1. In addition, *MIR126* stimulated lipid droplet accumulation in a hypoxia-inducible factor-1α (HIF1α)-dependent manner. *MIR126* also reduced pyruvate dehydrogenase kinase (PDK) and acetyl-CoA-citrate lyase (ACL) expression, leading to the accumulation of cytosolic citrate and paradoxical inhibition of pyruvate dehydrogenase (PDH) activity. Simultaneous pharmacological and genetic intervention with PDK and ACL activity phenocopied the effects of *MIR126.* This suggests that in MM *MIR126* initiates a metabolic program leading to high autophagic flux and HIF1α stabilization, incompatible with tumor progression of MM. Consistently, *MIR126*-expressing MM cells injected into immunocompromised mice failed to progress beyond the initial stage of tumor formation, showing that increased autophagy has a protective role in MM.

## INTRODUCTION

Metabolic reprogramming of cancer cells is essential for their adaptation to tumor microenvironment and for maintenance of tumor growth [1]. Autophagy is a catabolic pathway that has a fundamental role in this adaptation [2]. Depending on the context, autophagy can induce cell death or promote tumor inhibition [3]. Malignant transformation is frequently associated with suppression of autophagy. Recent implication of tumor suppressors like beclin-1 (BECN1) in autophagic pathways indicates a causative role for autophagy deficiencies in cancer formation. It has been documented that autophagy is strictly controlled to maintain homeostatic balance of energy metabolism and turnover of proteins as well as cellular organelles [4,5]. This process is post-transcriptionally regulated by small non-coding microRNAs (*MIRs*) that regulate gene expression via complementary base-pairing with mRNAs [6].

Among various *MIRs*, *MIR126* has an important role in cancer biology, since it can inhibit progression of certain cancers via negative control of proliferation, migration, invasion and cell survival [7,8]. In tumour cells, *MIR126* alters a number of cellular functions via suppressing translation of different target genes [9]. Anti-proliferative effect of *MIR126* was found in several tumour types including colon cancer, non-small cell lung cancer and malignant mesothelioma (MM) via targeting different members of the PI3K/AKT pathway [10–12]. Similarly, *MIR126* was found to suppress tumours by directly targeting the insulin receptor substrate-1 (IRS1) [12–14] and the disintegrin- and metalloproteinase domain-containing protein-9 (ADAM9) [15]. Using luciferase assay, it was found that *MIR126* targets other factors, such as SOX2, SLC7A5, EGFL7 and VEGF [16–20].

Here, we report that *MIR126* acts as an inducer of autophagic flux in MM. Ectopic overexpression of *MIR126* increased autophagic activity by altering the insulin signaling pathway through IRS1, resulting in reduced glucose uptake. Under low glucose conditions, MM cells activated the AMPK/mTOR signaling pathway as an energy-dependent regulator of autophagy. We also show that *MIR126* overexpression was accompanied by accumulation of intracellular lipid droplets (LDs) in MM cells due to alteration of mitochondrial function in a hypoxia-inducible factor-1α (HIF1α)-dependent manner.

## RESULTS

### Overexpression of *MIR126* induces autophagic flux

**Figure 1:**
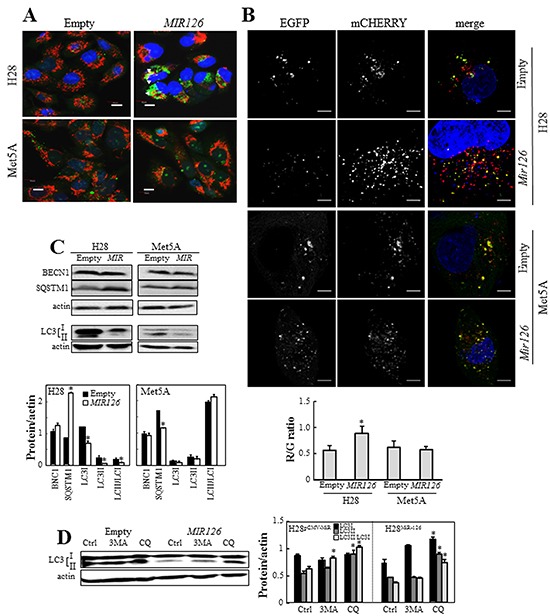
Ectopic *MIR126* induces autophagic flux **A.** *MIR126*-transfected H28 and Met5A cells and their empty plasmid-transfected counterparts were stained with AO and TMRM, and AVs (green) and mitochondria (red) visualized by fluorescent microscopy. The yellow spots indicate overlaying AVs and mitochondria. Scale bars represent 10 μm. **B.** *MIR126*-transfected H28 and Met5A cells and their empty plasmid-transfected counterparts were transiently transfected with dual fluorescence mCHERRY-EGFP-LC3B vector, and the red (R, lysosomal autophagosomes), green (G, autophagosomes) were visualized. Typical cells are shown, scale bars represent 5 μm. Evaluation of the ratio of red/green dots in empty plasmid- and *MIR126*-transfected H28 and Met5A cells. The number of cells evaluated in each condition was n=56 for H28 and 32 for Met5A, from 5 independent experiments. The symbol “*” indicates significant difference between mock and *MIR126* cells. Error bars indicate S.E.M. **C.** Expression of autophagic markers BECN1, SQSTM1, and LC3I/II. Densitometric evaluation of the bands shown in D related to the level of actin (lower panel). **D.** Overexpression of *MIR126* induces autophagic flux. *MIR126*-transfected Met5A and H28 cells and their empty plasmid-transfected counterparts were incubated in the presence and absence of 3MA or CQ for 24 h, and evaluated for LC3 conversion. Densitometric analysis of LC3 conversion related to actin (lower panel). The data shown are mean values ± S.D. derived from three independent experiments. Comparisons among groups were determined by one-way ANOVA with Tukey post-hoc analysis; the symbol “*” denotes significant differences compared with empty plasmid-transfected cells, or controls (Ctrl) versus treatments (3MA and CQ), with p < 0.05.

To explore the role of *MIR126* in autophagy, MM cells (cell line H28) and non-malignant mesothelial cells (cell line Met5A) transfected with *MIR126* and empty plasmid were stained with acridine orange (AO) or transfected with mCHERRY-EGFP-LC3B plasmid. Punctuate acid vesicle (AV) formation (AO staining) was evaluated by fluorescence microscope. As shown in Figure 1A, there was a marked increase of AVs in *MIR*-transfected MM cells compared to MM cells carrying an empty plasmid, which was reversed by blocking the function of *MIR126* using anti-*MIR126* (Supplementary Figure S1A upper panels). The AVs were perinuclear and overlayed with mitochondria suggesting a possible increase in autophagic/mitophagic flux in *MIR126*-expressing cells. In contrast, no effect on AV formation was found upon *MIR126* introduction into non-malignant Met5A cells. To directly assess the effect of *MIR126* on the autophagic flux, we transfected the *MIR126*-expressing and mock cells with a mCHERRY-EGFP-LC3B fusion construct. The LC3B-fused EGFP loses fluorescence due to the acidic environment of lysosomes, while mCHERRY retains it, discriminating between autophagosomes in the cytoplasm (visualized as yellow/green punctae) and those in the lysosome (visualized as red punctae). As shown in Figure 1B the ratio of red to green punctae was increased in *MIR126*-expressing malignant H28 cells indicating accelerated lysosomal delivery of autophagosomes and increased autophagic flux, but it remained unaffected by *MIR126* in non-malignant Met5A cells. This suggests that *MIR126* increases autophagic flux in MM cells.

Next, we assessed markers of autophagy including BECN1, SQSTM1 and the conversion of LC3I (cytosolic form) to LC3II (lipidated, autophagosome membrane-bound form) by western blotting (WB). *MIR126* overexpression led to significant upregulation of SQSTM1 in MM cells, while BECN1 did not show significant changes (Figure 1C). In cancer cells, *MIR126* induced reduction of LC3II levels rather than its accumulation, consistent with increased lysosomal delivery of the autophagosome-incorporated LC3II indicated by the dual fluorescence construct described above. To further determine whether *MIR126* induces autophagy flux or blocks autophagy initiation (this could also reduce the LC3II levels), we investigated LC3 turnover in the presence of autophagy inhibitors. Cells were treated with 3-methyladenine (3MA) or chloroquine (CQ) to block autophagosome formation, or autophagic degradation (autophagosome-lysosome fusion), respectively. CQ treatment caused significant increase of LC3II protein in *MIR126*-transfected MM cells, suggesting that the overexpression of *MIR126* indeed induces the autophagic flux. A slight effect in empty plasmid-transfected cells was also observed, indicating basal autophagic activity (Figure 1D). Inhibition of upstream steps of autophagy by 3MA also induced LC3II accumulation in MM cells. 3MA is widely used as autophagy inhibitor based on its inhibitory effect on class III PI3K activity. However, 3MA was found to promote autophagic flux under certain conditions [21]. It was reported that 3MA can induce autophagy similarly as rapamycin, via suppression of mTOR function [22]. It is therefore likely that LC3II accumulation in this situation is the result of 3MA-induced autophagy. Collectively, these results indicate that *MIR126* expression upregulates autophagic flux in MM cells but not in their non-malignant counterparts.

### Insulin receptor substrate-1 (IRS1), a target of *MIR126*, is involved in autophagic activity

IRS1 is the functional downstream target of *MIR126* via its 3′UTR [12–14]. IRS1, activated from the insulin-like growth factor-1 (IGF1) receptor, recruits intracellular proteins to transduce incoming signals in a cascade-like manner, leading to activation of the PI3K/mTOR signaling, negatively regulating autophagy. To evaluate the role of IRS1 in *MIR*126-induced autophagy, IRS1 was overexpressed or silenced in *MIR*- and empty vector-transfected MM cells, and AV formation and LC3 conversion were evaluated. As previously reported, *MIR126* downregulates IRS1 [12], which is associated with increased AV formation and reduced level of LC3II/LC3I ratio as a result of increased autophagic activity. Overexpression of IRS1 inhibited AV formation and induced LC3II accumulation in *MIR126* transfected cells. Conversely, IRS1 silencing induced AV formation and reduced LC3II level in both *MIR126*- and empty plasmid-transfected MM cells, thus resembling *MIR126* overexpression (Supplementary Figure 2). Additionally, we used IstMes2 cells with truncated IRS1 lacking its *MIR126* binding site (see the sequence in Figure S2) to address whether this finding also applies to unchanged IRS1 level. IstMes2 cells carrying truncated IRS1 were not sensitive to *MiR126*-induced autophagy (Supplementary Figure S3A, S3B). Inhibition of autophagy by 3MA or CQ did not induce further LC3II accumulation in *MIR126*- and empty plasmid-transfected IstMes2 cells, supporting the premise that these cells lack autophagic activity (Figure S3C). Conversely, suppression of IRS1 by siRNA induced AV formation and LCII turnover as a result of increased autophagic flux (Figure S3D, S3E).

**Figure 2:**
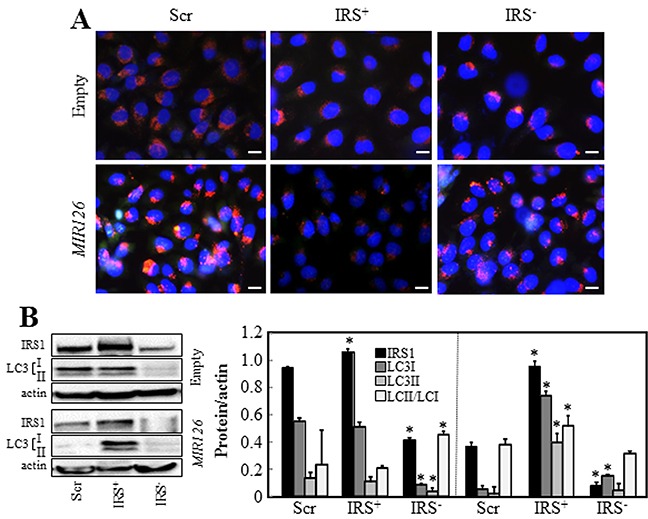
IRS1 is involved in *MIR126*-induced autophagy Empty plasmid- and *MIR126*-transfected H28 cells, and their IRS1-silenced (IRS1-) or IRS1-overexpressing (IRS1+) counterparts were analyzed for AV formation **A.** IRS1 levels and LC3 conversion **B.** Densitometric analysis of IRS1 and LC3 conversion related to actin (right panel). Scale bar for all images equals 10 μm. The data shown are mean values ± S.D. derived from three independent experiments. Comparisons among groups were determined by one-way ANOVA with Tukey post-hoc analysis; the symbol “*” indicates significantly different values compared with scramble control with p < 0.05.

### *MIR126* affects glucose uptake

**Figure 3:**
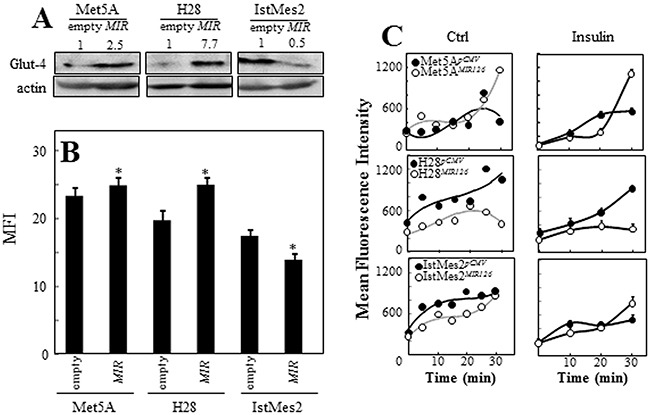
*MIR126* induces GLUT-4 expression and inhibits glucose uptake *MIR126*-transfected Met5A, H28 and IstMes2 cells and their empty plasmid-transfected counterparts were evaluated for GLUT-4 expression by WB **A.** and its cell surface expression by flow cytometry **B, C.** Glucose uptake was evaluated over time by 2-NBDG (50 μM) in low-glucose DMEM without and with insulin stimulus, and expressed as mean fluorescent intensity (MFI). The data shown are mean values ± S.D. derived from five independent experiments. The symbol “*” indicates significant differences compared with empty plasmid-transfected cells.

*MIR126* may affect the insulin pathway signaling by targeting IRS1. We therefore asked whether *MIR126* alters glucose homeostasis in the context of altered autophagic response. The glucose transporter-4 (GLUT-4) is the main glucose carrier responsible for insulin-regulated glucose uptake [23]. As shown in Figure 3A, 3B, ectopic *MIR126* increased GLUT-4 expression in Met5A cells and, to a larger extent, in malignant H28 cells. On the other hand, decreased GLUT-4 levels were found in *MIR126*-non-responsive IstMes2 cells. Even though GLUT-4 was highly expressed in *MIR126*-transfected H28 cells, glucose uptake was markedly reduced in these cells also after insulin stimulus. Anti-*MIR126* transfection restored glucose uptake in these cells (Supplementary Figure S1B). However, the high level of GLUT-4 found in *MIR*-transfected Met5A paralleled high glucose uptake. No significant changes in glucose uptake were observed in *MIR126*-non-responsive IstMes2 cells (Figure 3C).

### *MIR126* regulates autophagy

**Figure 4:**
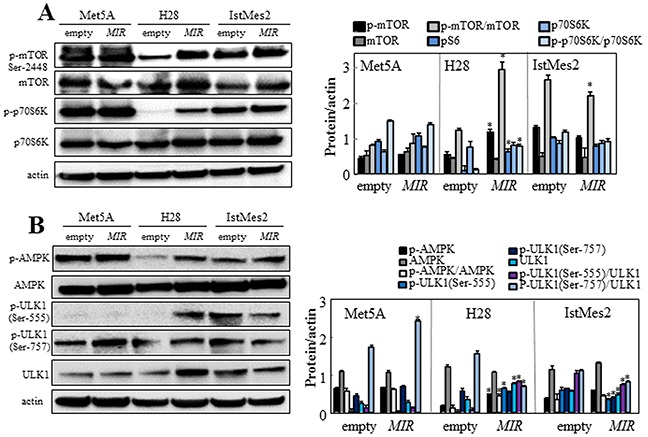
*MIR126* affects the mTOR/AMPK/ULK1 pathway *MIR126*-transfected Met5A, H28 and IstMes2 cells and their empty plasmid-transfected counterparts were analyzed for relative levels of p-mTOR, mTOR and p-p70S6K, p70S6K **A.** and for p-AMPK, AMPK, p-ULK1 (Ser555), p-ULK1 (Ser757) and ULK1 **B.** Densitometric evaluation of the bands shown relative to actin (right panels). The data shown are mean values ± S.D. derived from five independent experiments. The symbol “*” indicates significant differences compared with empty plasmid-transfected cells.

Limited availability of intracellular glucose can trigger autophagy. AMPK/mTOR signaling pathway, a major regulator of autophagy, plays a role in inhibition of autophagy via phosphorylation of UNC-51-like kinase 1 (ULK1), a component involved in initiation of the autophagic process. We evaluated the autophagic signaling pathway in *MIR126*- and empty plasmid-transfected cells. Ectopic *MIR126* increased mTOR expression in H28 cells but not in Met5A and IstMes2 cells. The p-mTOR/mTOR ratio, which indicates activation of mTOR, increased in H28 cells overexpressing *MIR126* (Figure 4A). Similarly, phosphorylation of the p70 ribosomal protein S6 kinase-1 (p70S6K), a downstream substrate of mTOR, was also increased in *MIR126*-transfected H28 cells. Overexpression of *MIR126* induced the AMPK phosphorylation, which in turn activated the ULK1 pathway. Increased phosphorylation of ULK1 at Ser-555 (pro-autophagic) and decreased phosphorylation at Ser-757 (anti-autophagic) were found in *MIR*-transfected H28 cells, but not in non-malignant Met5A cells and *MIR126*-non-responsive IstMes2 cells (Figure 4B). Restoration of the autophagic pathway was observed by blocking the function of *MIR126* using antisense *MIR126* (anti-*MIR*) (Figure S1C), further confirming the involvement of *MIR126*.

### Ectopic *MIR126* induces lipid accumulation in malignant cells

**Figure 5:**
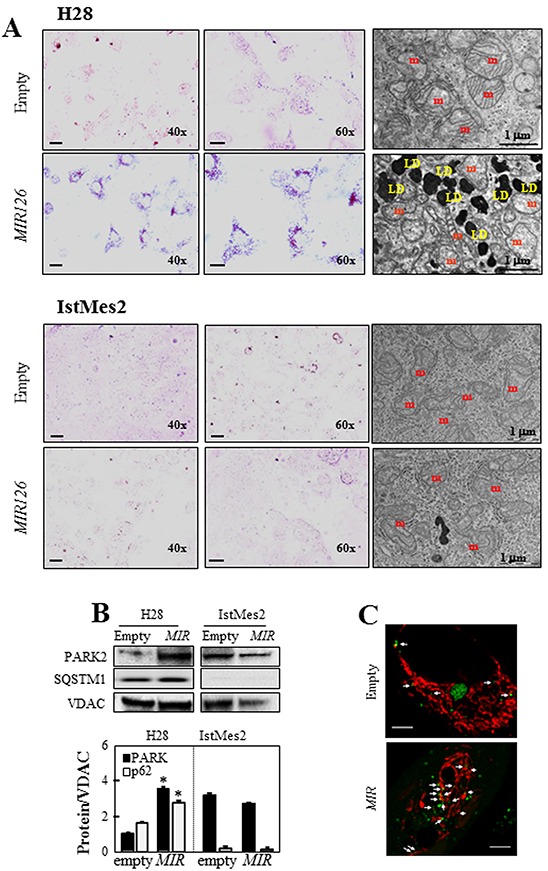
*MIR126* overexpression results in lipid accumulation **A.** H28 and IstMes2 cells transfected with empty plasmid and *MIR126* were subjected to Oil Red O staining (left panel), and TEM (right panel). Magnification/scale bar are shown in individual images; m = mitochondria, LD = lipid droplet. **B.** Immunoblot of PARK2, SQSTM1, and cytochrome c (cyt c) in mitochondria isolated from *MIR126*-transfected H28 and IstMes2 cells and their empty plasmid-transfected counterparts. Densitometric analysis related to voltage-dependent anion channel (VDAC) is shown in the bottom panel. **C.** EOS-LC3 co-localization with mitochondria stained with MitoTracker Far Red in empty- and *MIR126*-transfected H28 cells. The LC3 punctae decorating mitochondria are indicated by white arrows. Scale bar equals 5 μm. The data shown are mean values ± S.D. derived from three independent experiments. The symbol “*” indicates significant differences compared with empty plasmid-transfected cells.

We observed vesicle-like particles in the cytoplasm of *MIR126*-transfected H28 cells. Staining with Oil Red O revealed that the vesicles contain lipid droplets (LDs) (Figure 5A, left panels). The LD formation in *MIR126*-transfected H28 cells was inhibited by anti-MIR transfection (Figure S1A lower panel). This finding was supported by TEM (Figure 5A, right panels). Interestingly, mitochondria showed altered morphology, such as swollen appearance, which was not observed in control cells. We next investigated markers of mitophagy in *MIR126*-transfected cells. As shown in Figure 5B, ectopic *MIR126* induced PARK2 and SQSTM1accumulation in isolated mitochondria of H28 cells, indicating mitophagy. Mitophagy was confirmed in empty- and *MIR*-transfected H28 cells by EOS-LC3 co-localization with MitoTracker-stained mitochondria. As shown in Figure 5C, the LC3 punctae co-localized with mitochondria, being indicative of mitophagic process. Formation of LDs and presence of damaged mitochondria/mitophagy were not observed in *MIR126*-non-responsive IstMes2 cells (Figure 5).

Previous work demonstrated activation and stabilization of hypoxia-inducible factor-1α (HIF1α) in *MIR126*-transfected MM cells [12]. To assess whether *MIR126*-induced LDs and autophagy/mitophagy could be attributed to HIF1α, the hypoxia factor was silenced in *MIR126*-transfected H28 cells. A marked increase of LD accumulation was observed in parental cells with silenced HIF1α, while disappearance of LDs was found in their *MIR126*-transfected counterparts (Figure S4, left panels). HIF1α did not affect AV formation and the autophagy signaling pathway (Figure S4A, right panels). Conversely, increased GLUT-4 and PARK2 induced by *MIR126* was reversed by HIF1α -silencing, pointing to a HIF-dependent mechanism (Supplementary Figure S4B).

### *MIR126* induces autophagy by inhibiting PDK and ACL

A global picture of modulation of signaling pathways by *MIR126* was investigated by genome-wide gene expression analysis. We utilized the pathway enrichment analysis to compare *MIR126*-transfected cells with their empty vector-transfected counterparts (Figure S5). Ectopic *MIR126* regulated a number of genes, affecting different signaling pathways. These include various pathways involved in cancer, such as the MAPK pathway, DNA synthesis and repair, IGF regulation, angiogenesis, or the Wnt pathway, as well as pathways regulating senescence and autophagy (Supplementary Figure S5A).

**Figure 6:**
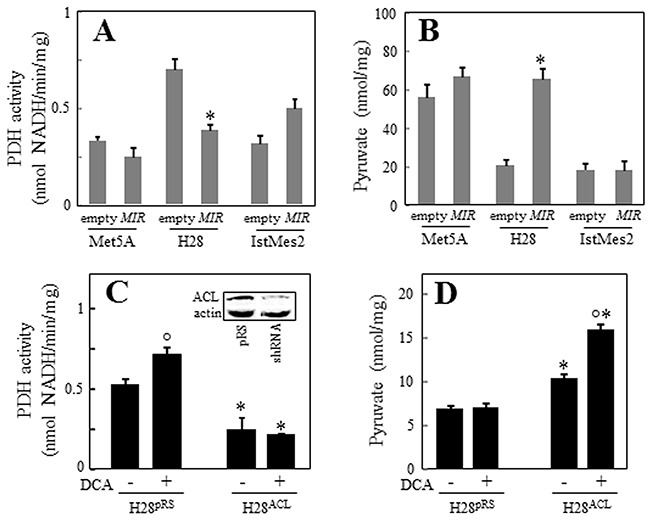
ACL and PDK knock-down increase pyruvate levels and affect PDH activity *MIR126* transfected Met5A, H28 and IstMes2 cells and their empty plasmid-transfected counterparts were analyzed for PDH activity **A.** and pyruvate levels **B.** H28 cells were selected to stably express the empty vector (H28^pRS^) or the ACL shRNA construct (H28^ACL^, insert), and PDH activity **C.** and pyruvate levels **D.** evaluated in the presence or absence of PDK inhibitor DCA (20 mM). The data shown are mean values ± S.D. derived from three independent experiments. Comparisons among groups were determined by one-way ANOVA with Tukey post-hoc analysis; the symbol “*” indicates significant differences compared with empty plasmid-transfected cells, and the symbol “°” indicates significance between DCA treated and untreated cells.

**Figure 7:**
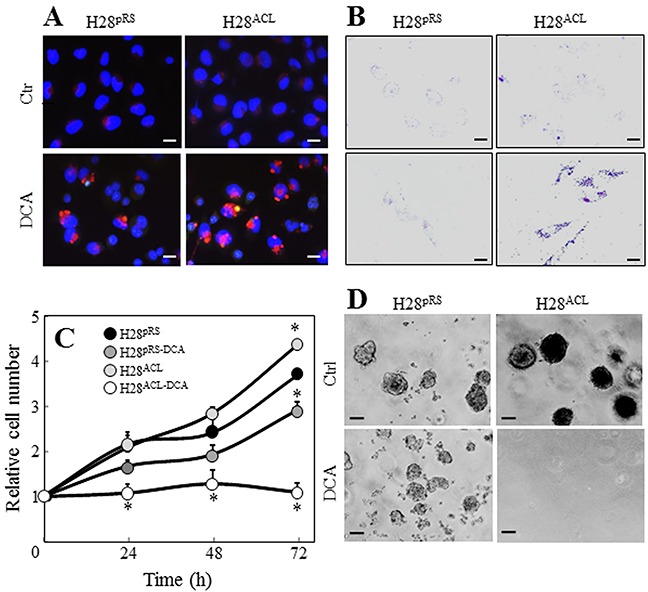
ACL and PDK knock-down promote autophagy and inhibit cell proliferation Cells transfected with empty vector (H28^pRS^) or ACL shRNA construct (H28^ACL^) were evaluated for AV **A.** and LD **B.** formation, and for cell proliferation **C.** and colony-forming activity **D.** in the presence and absence of DCA (20 mM). Images are representative of three independent experiments. Scale bars for cell and soft agar images are 10 μm and 100 μm, respectively. Comparisons among groups were determined by one-way ANOVA with Tukey post-hoc analysis; the symbol “*” indicates significant differences compared with empty vector-transfected cells.

**Figure 8:**
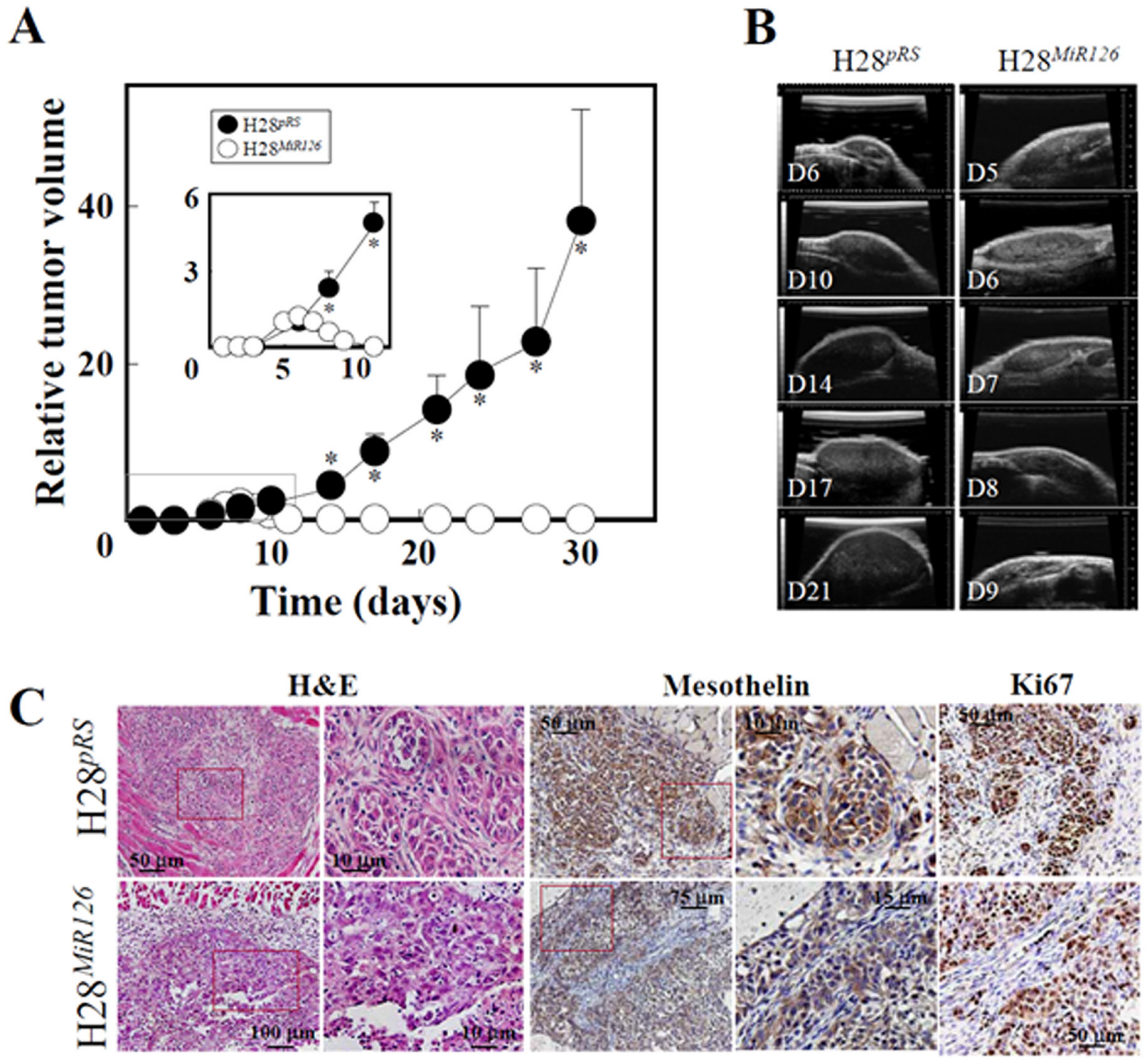
*MIR126* suppresses tumor formation **A.** Balb-c *nu/nu* mice were injected subcutaneously with 1×10^6^ H28^pRS^ or H28^MiR126^ cells and tumor growth was quantified by USI. The inset shows details of tumor size in the initial stage of the experiment. The results are derived from 6 mice in each group, and the symbol ‘*’ indicates significant differences with *p*<0.05. **B.** Shown here are representative images of tumors derived from the two H28 sublines acquired on individual days. **C.** Tumors from day 6 were sectioned and stained with H&E, as well as subjected to immunohistochemistry using anti-mesothelin IgG to identify MM cells in the sections and anti-Ki67 IgG to see the level of proliferation of tumor cells. H&E staining clearly documents lack of ‘structure’ of the H28^MiR126^ cell-derived tumors, which is obvious in tumors derived from cells transfected with the empty plasmid.

Gene array analysis revealed that one of the most profoundly downregulated genes in *MIR126*-transfected H28 cells was pyruvate dehydrogenase kinase (*PDK*) (Supplementary Figure S5B). The PDK protein inhibits mitochondrial pyruvate dehydrogenase (PDH), which catalyses irreversible decarboxylation of pyruvate to acetyl-CoA (AcCoA). Paradoxically, downregulation of PDK by *MIR126* resulted in reduction of PDH activity associated with an increase of its substrate, pyruvate, in malignant H28 cells (Figure 6A, 6B). In our previous work, we reported that *MIR126* increased cellular citrate by inhibiting the ACL activity [12]. Therefore, to evaluate the role of PDK and ACL in the mediation of *MIR126* effect, H28 cells were silenced for ACL activity, and PDK was inhibited by treatment with dichloroacetate (DCA). As shown in Figure 6C, DCA applied as a single agent induced PDH activity, consistent with its PDK-inhibitory role. Interestingly, ACL silencing markedly reduced PDH activity, which was also observed in DCA-treated H28 cells. In these cells, pyruvate levels increased (Figure 6D), thus resembling *MIR126*-transfected H28 cells with low PDH activity and high pyruvate level. These metabolic changes resulted in AV formation and LD accumulation, which were associated with inhibition of cell proliferation and colony-forming activity (Figure 7A-7D). Since these effects of *MIR126* in H28 cells are consistent with its tumor suppressor properties, we tested its effect on tumor growth. Figure 8 documents that while H28^pRS^ mock cells progressively formed tumors, H28*^MIR126^ MIR126*-transfected cells formed small tumors that were resorbed by day 10, reflecting the anti-tumor efficacy of *MIR126 in vivo*.

## DISCUSSION

*MIR126* has been reported to suppress progression of MM by affecting cellular metabolism [12]. Here, we found that *MIR126* is a potent inducer of autophagy in MM cells. We discovered that ectopic *MIR126-*induced massive AV formation and increased autophagic flux via autophagosome/lysosome fusion, as indicated by increased LC3II turnover and increased delivery of LC3 into lysosomes (*cf* Figure 1). This *MIR126*-induced autophagic process is mediated by IRS1. Down-regulation of IRS1 by *MIR126* or silencing of IRS1 reduced the LC3II/LC3I ratio as a result of high autophagic activity, which was reversed by IRS1 overexpression (*cf* Figure 2). IstMes2 MM cells lacking the *MIR126*-binding site within the IRS1 3′UTR further confirmed the role of IRS1 (*cf* Supplementary Figure S3).

Insulin receptor substrates (IRS), critical components of insulin signaling, are involved in cell proliferation, metabolism, and cancer development [24]. It was reported that IRS block basal autophagy via inhibition of PI3K/mTOR signaling [25]. Contrary to our expectation, *MIR126* induced activation of the mTOR pathway in MM cells. *MIR126*-transfected H28 cells also showed activated AMPK/ULK1 pathway, a sensor of intracellular energy homeostasis [26] (*cf* Figure 4), suggesting that the AMPK/ULK1 pathway takes precedence in autophagy regulation upon *MIR126* expression in MM cells. The increased AMPK activity could be related to reduced glucose uptake observed for *MIR126* H28 cells, which was independent of GLUT-4. Hence, the increased GLUT-4 in this scenario could result from compensatory upregulation driven by HIF1α that we previously found to be increased by *MIR126* in MM cells [12]. In contrast, in non-malignant mesothelial cells, increased expression of GLUT-4 by *MIR126* was associated with higher glucose uptake, pointing to very distinct effects of *MIR126* in MM and non-malignant cells.

Surprisingly, *MIR126* induced ~50-fold downregulation of PDK in MM cells, yet decreased PDH activity, even though an increase of PDH activity would be expected in this situation. PDK is a negative regulator of the mitochondrial PDH complex, which modulates the balance between oxidation of glucose and lipids, depending on the nutritional status, and plays role of metabolic switch for fuel selection [27]. Clinical evidence has shown that upregulation of PDK correlates with advanced malignancy [28, 29], and its inhibition significantly suppresses tumor growth in mouse xenograft models [30, 31]. Although ectopic *MIR126* inhibited PDK, PDH activity was reduced in *MIR126*-transfected H28 cells compared to their empty-plasmid counterparts.

Previously we have found that *MIR126* upregulation in MM cells decreased ACL activity, inducing citrate accumulation in the cytoplasm and stabilization of HIF1α [12]. ACL is responsible for the conversion of citrate to cytosolic AcCoA, an important component of several biosynthetic pathways. In addition, cytosolic AcCoA can regulate autophagy by protein acetylation [32, 33]. In this study, we found that simultaneous inhibition of PDK and ACL by pharmacological and genetic means recapitulated the effects of *MIR126* on tumorigenic potential and autophagy, while single treatment had little or no effect. This means that PDK and ACL together are principal effectors of *MIR126* response initiated at IRS1, which regulates ACL via AKT [12].

An interesting finding of this study is that ACL inhibition (by *MIR126* or by genetic intervention) can override the removal of PDK-mediated control and maintain an inactive PDH complex. While the precise nature of this interesting phenomenon is not clear, it could be related to increased mitochondrial reducing activity and limited mitochondrial respiration previously reported in *MIR126* MM cells [12], and reflect direct inhibition of PDH complex by increased level of mitochondrial NADH. In addition, mitochondria compromised by *MIR126* in conjunction with higher autophagic flux instigate increased mitophagy observed in this study for *MIR126*-expressing MM cells.

The accumulation of cytoplasmic lipids in LDs found in *MIR126*-transfected MM cells could result from direct replenishment of LD linked to increased autophagy [34], or it could relate to the elevated HIF1α we previously identified in *MIR126*-expressing MM cells [12]. We consider the second option more likely, as we show that HIF1α knock down did not affect autophagy in *MIR126* MM cells, but eliminated LD formation. Therefore, not autophagy, but direct HIF1α regulation, for example via hypoxia-inducible protein 2 (HIG2) which is a down-stream target of HIF1α implicated in LD biogenesis [35], is responsible for LD formation upon *MIR126* expression.

**Figure 9:**
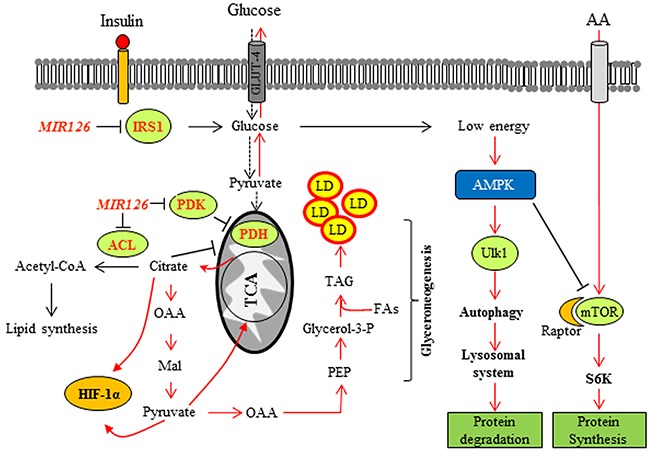
Proposed model of *MIR126* in regulating autophagy and cell proliferation *MIR126* targets the expression of IRS1 resulting in the insulin signaling pathway disruption. This contributes to lower glucose uptake. Upon energy depletion, the AMPK pathway is activated and induces lysosomal autophagy. Inhibition of ACL by *MIR126* contributes to citrate accumulation in cytosol, inducing HIF1α activation and stabilization, leading to GLUT-4 upregulation. *MIR126* induces PDK down-regulation, which in conjunction with ACL inhibition paradoxically inhibits PDH, possibly due to the high level of NADH and citrate/pyruvate in mitochondrial matrix. The associated HIF1α stabilization results in lipid droplets accumulation (LD) via increased ‘glyceroneogenesis’ [12]. This complex metabolic program leads to the loss of malignancy in MM. The activated pathways are shown in red. OAA, oxaloacetic acid; Mal, malic acid; PEP, phosphoenolpyruvate.

In summary, we show that *MIR126* induces complex metabolic reprogramming of MM cells including activation of the autophagic pathway following disruption of IRS signaling, downregulation of PDK and ACL activity, accumulation of citrate, and formation of LDs in the cytoplasm in a HIF1α-dependent manner (Figure 9). Collectively, these effects inhibit tumor progression when *MIR126* is high, emphasizing the protective role of enhanced autophagic flux in the progression of MM.

## MATERIALS AND METHODS

### Cell culture and treatments

Non-malignant mesothelial cells (Met5A) and sarcomatoid MM cells (H28) were obtained from the ATCC. Immortalized IstMes2 cells (epithelioid) were established from a patient and were identified morphologically by a phenotypic analysis [36]. All cell lines were grown in the RPMI-1640 medium with antibiotics and 10% FBS. Cells were treated for 24 h with 3-methyladenine (3MA, 50 μM) or chloroquine (CQ, 10 μM) to block autophagosome formation or autophagic degradation, respectively, and with dichloroacetate (DCA, 20 mM) to inhibit PDK activity.

### *MIR126*, siRNA and transfections

Cells (2×10^5^) were stably transfected with 1 μg of the pCMV-MiR and/or pRS (OriGene) empty plasmid or with plasmid carrying the *MIR126* sequence 5′-UCG UAC CGU GAG UAA UAA UGC G-3′ (OriGene, Rockville, MD) and/or shRNA pRS plasmid, with the HIF1α-targeting sequence of 5′-ACA AGA ACC TAC TGC TAA TGC CAC CAC TA-3′, and with the acetyl-CoA citrate lyase (ACL)-targeting sequence of 5′-CCA TCA CTG AGG TCT TCA AGG AAG AGA TG-3′, using the TransIT-LT1 reagent (Mirus). Selection was carried out with G418 (Sigma) and/or puromycin (Sigma) added to the cell culture media after transfection at 0.6 mg/ml and 1 μg/ml for 48 h, respectively. G418- and/or puromycin-resistant clones were analyzed for *MIR126* and HIF1α expression. Selected clones were maintained in RPMI with 0.6 mg/ml G418 and/or 1 μg/ml puromycin. Anti-IRS1 sequence 5′-AGA CCA UCA GCU UCG UGA ATT-3′ (Ambion), or human IRS1 plasmid (SC124032, OriGene), were used to transiently silence or overexpress IRS1, respectively. *MIR126* function was blocked with the anti-sense oligonucleotide 5′-GCA UUA UUA CUC ACG GUA CGA-3′ (IDT, Tema Ricerca). Scrambled sequences were used as a control. Cells (2×10^5^) were transfected with the oligonucleotide (1 μg/well) using TransIT-LT1. After 48 h, the levels of IRS1 mRNA were evaluated by WB analysis.

### Transfection with EOS-LC3 and EGFP-mCHERRY-LC3B vectors, image acquisition and analysis

*MIR126*-transfected cells (Met5A, H28) and their empty-plasmid counterparts were transiently transfected with the pLENTI6.3 EOS-LC3 expression plasmid (a kind gift from Dr. P. Jezek, Institute of Physiology, CAS) or the dual fluorescence pBabe-puro-mCHERRY-EGFP-LC3B vector [50] using Lipofectamine 3000 (Life Technologies) two days before the experiment in glass bottom microscopy dishes (In Vitro Scientific). For EOS-LC3 imaging, the mitochondria were stained with MitoTracker Far Red (20 nM, 15 min; Life Technologies), and for mCHERRY-EGFP-LC3 imaging, the cell nuclei were stained with Hoechst33342 (200 ng/ml, 15 min; Sigma). Images of live cultures were captured by Leica SP5 confocal microscope using HCX PL APO 63x/1,30 glycerol immersion objective, zoom 5, scanning speed 1,000 Hz and line averaging 2 at 37°C and 5% CO_2_. Images were deconvoluted with the Huygens professional software package (SVI). The scoring for yellow/green (autophagosomes) and red dots (autophagosomes inside lysosomes) produced by the dual fluorescence mCHERRY-EGFP- LC3B vector was done manually in ImageJ. The dots were scored as red only when no signal was detected in the green channel.

### Acid vesicles (AVs) and mitochondrial staining

For detection of AVs, cells were seeded on cover-slips, allowed to attach overnight, and incubated with 5 μg/ml AO (Sigma) for 30 min at 37°C. Mitochondria were visualized with 200 nM tetramethyl rhodamine methyl ester (TMRM, Invitrogen). Cells were analyzed using confocal microscopy (Zeiss, Axiocam MRc5; magnification 40 x or 60x).

### Western blot analysis

Cells or isolated mitochondria were lysed in the RIPA buffer containing Na_3_VO_4_ (1 mM) and protease inhibitors (1 μg/ml). The cell lysate proteins were separated using SDS-PAGE and transferred onto nitrocellulose membranes (Protran). After blocking with 5% non-fat milk in PBS-Tween (0.1%), the membranes were incubated with antibodies against IRS1, HIF1α (both Bethyl), BECN1, SQSTM1/p62, LC3B, phosphor-p70S6K, phospho-AMPK, AMPK, phospho-ULK1 (Ser-555), phospho-ULK1 (Ser-757), and ULK1 (all Cell Signaling), phospho-mTOR (p-mTOR, Ser-2448), mTOR, PARK2 (all ThermoFisher Scientific), and GLUT-4, cytochrome c and VDAC1 (all Santa Cruz). After incubation with the HRP-conjugated secondary IgG (Sigma), blots were developed using the ECL detection system (Pierce Biotechnology). The band intensities were visualized and quantified with ChemiDoc using the Quantity One software (BioRad Laboratories).

### Glucose uptake

Cells were seeded in 96-well black-bottom plates (2×10^4^ cells per well) and incubated in DMEM with low glucose (1 g/l) overnight at 5% CO_2_ and 37°C. The cells were then incubated for 30 min with 50 μM 2-nitrobenzodeoxyglucose (2-NBDG) in low-glucose DMEM without or with insulin (50 μg/ml). The level of fluorescent glucose analogue in the cells was evaluated at 550/590 nm using a fluorescence plate reader (Infinite F200 PRO, Tecan).

### Oil Red O staining of lipids

Cells grown on coverslips were fixed in 70 % (v/v) cold ethanol before staining with Oil Red O solution in 60% (v/v) isopropanol. For visualization, bright-field images were captured at 40x and 63x magnification using the Axiocam MRc5 fluorescence microscope (Zeiss).

### Transmission electron microscopy (TEM)

Cells were fixed in 2.5% glutaraldehyde in phosphate-buffered saline (PBS) for 4 h and centrifuged to form pellets. The pellets were then rinsed in 0.1 M cacodylate buffer (Electron Microscopy Sciences), post-fixed in 1% osmium tetroxide for 30 min at 4°C, processed with propylene oxide, and embedded in mixture of Epon-Araldite. Thin sections were obtained with an LKB NOVA ultratome (Bromma), stained with lead citrate, and examined using the Philips CM 10 TEM.

### Soft agar colony formation

Cells were seeded in 0.7% low melting point agar in 24-well plates, overlaid with 0.35% low melting point agar, and cultured at 37°C in 5% CO_2_ for 1 month. Every 7 days, 0.5 ml of fresh medium was added to each well and the number of colonies counted.

### Isolation of mitochondria

Mitochondria were isolated from H28 and IstMes2 *MIR126*-transfected cells and their empty plasmid-transfected counterparts using a commercial kit (Sigma) according to the manufacturer's instructions. The results were normalized to the total protein determined using the Bradford assay (Sigma).

### Evaluation of pyruvate and pyruvate dehydrogenase (PDH) activity

Intracellular pyruvate and PDH activity were evaluated using a colorimetric kit (Sigma) according to the manufacturer's protocol. The results were normalized to the total protein.

### Gene expression microarray

Gene expression was assessed using the Agilent 44K microarray technology. Total RNA from *MIR126*- and empty plasmid-transfected H28 cells was isolated using the RNeasy kit (Qiagen) following the manufacturer's protocols. Total RNA (1 μg) was converted to labelled cRNA with nucleotides coupled to a fluorescent dye (Cy3) using the QuickAmp Kit (Agilent Technologies). Universal RNA from Invitrogen was labeled with Cy5 as a reference. Samples were purified using the RNeasy kit and quantified for dye integration using Nanodrop-8000 (Thermo Scientific). Following quantification, samples were hybridized overnight in a rotating hybridization oven and washed/scanned using an Agilent scanner. Microarrays were processed by normexp background correction and loess normalization. Pathways enrichment analysis was performed using Gene Spring software version 12.

### Animal experiments

Balb-c *nu/nu* mice were injected subcutaneously with 1×10^6^ H28^pRS^ or H28^MiR126^ cells per animal resuspended in 100 μl. Tumor volume was monitored by the ultrasound imaging (USI) instrument Vevo770 (VisualSonics) as described previously [37]. Each experimental group contained 6 mice. On day 6-7, representative tumors were excised from euthanized mice, paraffin-embedded, sectioned and stained with hematoxylin/eosin (H&E) as well as subjected to immunohistochemistry using anti-mesothelin IgG and anti-Ki67 IgG, following a standard procedure. All experiments were approved by the Griffith University Ethics Committee and performed according to the guidelines of the Australian and New Zealand Council for the Care and Use of Animals in Research and Teaching.

### Statistics

Results are expressed as mean ± S.D. unless indicated otherwise. Comparisons among groups of data were made using one-way ANOVA with Tukey post hoc analysis. The two-tailed Student's t-test was used to compare two groups. Differences with p<0.05 were considered statistically significant. All data generated in this study were analyzed using the SPSS software.

## SUPPLEMENTARY FIGURES



## References

[1] Cairns RA, Harris IS, Mak TW (2011). Regulation of cancer cell metabolism. Nat Rev Cancer.

[2] Liu H, He Z, Simon H (2015). Protective role of autophagy and autophagy-related protein 5 in early tumorigenesis. J Mol Med.

[3] Gozuacik D, Kimchi A (2004). Autophagy as a cell death and tumor suppressor mechanism. Oncogene.

[4] Green DR, Galluzzi L, Kroemer G (2014). Metabolic control of cell death. Science.

[5] Kenific CM, Debnath J (2015). Cellular and metabolic functions for autophagy in cancer cells. Trends Cell Biol.

[6] Kim KM, Kim SG (2014). Autophagy and microRNA dysregulation in liver diseases. Arch Pharm Res.

[7] Ebrahimi F, Gopalan V, Smith RA, Lam AK (2014). MiR-126 in human cancers: clinical roles and current perspectives. Exp Mol Pathol.

[8] Tai HC, Chang AC, Yu HJ, Huang CY, Tsai YC, Lai YW, Sun HL, Tang CH, Wang SW (2014). Osteoblast-derived WNT-induced secreted protein 1 increases VCAM-1 expression and enhances prostate cancer metastasis by down-regulating miR-126. Oncotarget.

[9] Chakraborty C, Sharma AR, Patra BC, Bhattacharya M, Sharma G, Lee SS (2016). MicroRNAs mediated regulation of MAPK signaling pathways in chronic myeloid leukemia. Oncotarget.

[10] Guo C, Sah JF, Beard L, Willson JK, Markowitz SD, Guda K (2008). The noncoding RNA, miR-126, suppresses the growth of neoplastic cells by targeting phosphatidylinositol 3-kinase signaling and is frequently lost in colon cancers. Genes Chromosomes Cancer.

[11] Chen SW, Wang TB, Tian YH, Zheng YG (2015). Down-regulation of microRNA-126 and microRNA-133b acts as novel predictor biomarkers in progression and metastasis of non small cell lung cancer. Int. J. Clin. Exp. Pathol..

[12] Tomasetti M, Nocchi L, Staffolani S, Manzella N, Amati M, Goodwin J, Kluckova K, Nguyen M, Strafella E, Bajzikova M, Peterka M, Lettlova S, Truksa J (2014). MicroRNA-126 suppresses mesothelioma malignancy by targeting IRS1 and interfering with the mitochondrial function. Antioxid Redox Signal.

[13] Zhang J, Du YY, Lin YF, Chen YT, Yang L, Wang HJ, Ma D (2008). The cell growth suppressor, mir-126, targets IRS-1. Biochem Biophys Res Commun.

[14] Ryu HS, Park SY, Ma D, Zhang J, Lee W (2011). The induction of microRNA targeting IRS-1 is involved in the development of insulin resistance under conditions of mitochondrial dysfunction in hepatocytes. PLoS One.

[15] Felli N, Felicetti F, Lustri AM, Errico MC, Bottero L, Cannistraci A, De Feo A, Petrini M, Pedini F, Biffoni M, Alvino E, Negrini M, Ferracin M (2013). MiR-126 & 126* restored expressions play a tumor suppressor role by directly regulating ADAM9 and MMP7 in melanoma. PLoS One.

[16] Hara T, Jones MF, Subramanian M, Li XL, Ou O, Zhu Y, Yang Y, Wakefield LM, Hussain SP, Gaedcke J, Ried T, Luo J, Caplen NJ (2014). Selective targeting of KRAS-mutant cells by miR-126 through repression of multiple genes essential for the survival of KRAS-mutant cells. Oncotarget.

[17] Yang C, Hou C, Zhang H, Wang D, Ma Y, Zhang Y, Xu X, Bi Z, Geng S (2013). MiR-126 functions as a tumor suppressor in osteosarcoma by targeting Sox2. Int J Mol Sci.

[18] Miko E, Margitai Z, Czimmerer Z, Várkonyi I, Dezso B, Lányi A, Bacsó Z, Scholtz B (2011). MiR-126 inhibits proliferation of small cell lung cancer cells by targeting SLC7A5. FEBS Lett.

[19] Sun Y, Bai Y, Zhang F, Wang Y, Guo Y, Guo L (2010). MiR-126 inhibits non-small cell lung cancer cells proliferation by targeting EGFL7. Biochem Biophys Res Commun.

[20] Chen H, Li L, Wang S, Lei Y, Ge Q, Lv N, Zhou X, Chen C (2014). Reduced miR-126 expression facilitates angiogenesis of gastric cancer through its regulation on VEGF-A. Oncotarget.

[21] Wu YT, Tan HL, Shui G, Bauvy C, Huang Q, Wenk MR, Ong CN, Codogno P, Shen HM (2010). Dual role of 3-methyladenine in modulation of autophagy via different temporal patterns of inhibition on class I and III phosphoinositide 3-kinase. J Biol Chem.

[22] Meijer AJ, Codogno P (2009). Autophagy: regulation and role in disease. Crit Rev Clin Lab Sci.

[23] Marín-Juez R, Diaz M, Morata J, Planas JV (2013). Mechanisms regulating GLUT4 transcription in skeletal muscle cells are highly conserved across vertebrates. PLoS One.

[24] Geng Y, Ju Y, Ren F, Qiu Y, Tomita Y, Tomoeda M, Kishida M, Wang Y, Jin L, Su F, Wei C, Jia B, Li Y, Chang Z (2014). Insulin receptor substrate 1/2 (IRS1/2) regulates Wnt/β-catenin signaling through blocking autophagic degradation of disheveled-2. J Biol Chem.

[25] Chan SH, Kikkawa U, Matsuzaki H, Chen JH, Chang WC (2012). Insulin receptor substrate-1 prevents autophagy-dependent cell death caused by oxidative stress in mouse NIH/3T3 cells. J Biomed Sci.

[26] Mihaylova MM, Shaw RJ (2011). The AMPK signalling pathway coordinates cell growth, autophagy and metabolism. Nat Cell Biol.

[27] Sugden MC, Bulmer K, Holness MJ (2001). Fuel-sensing mechanisms integrating lipid and carbohydrate utilization. Biochem Soc Trans.

[28] Koukourakis MI, Giatromanolaki A, Sivridis E, Gatter KC, Harris AL (2005). Pyruvate dehydrogenase and pyruvate dehydrogenase kinase expression in non-small cell lung cancer and tumor-associated stroma. Neoplasia.

[29] Lu CW, Lin SC, Chien CW, Lee CT, Lin BW, Lee JC, Tsai SJ (2011). Overexpression of pyruvate dehydrogenase kinase 3 increases drug resistance and early recurrence in colon cancer. Am J Pathol.

[30] McFate T, Mohyeldin A, Lu H, Thakar J, Henriques J, Halim N D, Wu H, Schell MJ, Tsang TM, Teahan O, Zhou S, Califano JA, Jeoung NH (2008). Pyruvate dehydrogenase complex activity controls metabolic and malignant phenotype in cancer cells. J Biol Chem.

[31] Moore JD, Staniszewska A, Shaw T, D'Alessandro J, Davis B, Surgenor A, Baker L, Matassova N, Murray J, Macias A, Brough P, Wood M, Mahon PC (2014). VER-246608, a novel pan-isoform ATP competitive inhibitor of pyruvate dehydrogenase kinase, disrupts Warburg metabolism and induces context-dependent cytostasis in cancer cells. Oncotarget.

[32] Baggetto LG (1992). Deviant energetic metabolism of glycolytic cancer cells. Biochimie.

[33] Schroeder S, Pendl T, Zimmermann A, Eisenberg T, Carmona-Gutierrez D, Ruckenstuhl C, Mariño G, Pietrocola F, Harger A, Magnes C, Sinner F, Pieber TR, Dengjel J (2014). Acetyl-coenzyme A: a metabolic master regulator of autophagy and longevity. Autophagy.

[34] Rambold AS, Cohen S, Lippincott-Schwartz J (2015). Fatty acid trafficking in starved cells: regulation by lipid droplet lipolysis, autophagy, and mitochondrial fusion dynamics. Dev Cell.

[35] Gimm T, Wiese M, Teschemacher B, Deggerich A, Schödel J, Knaup KX, Hackenbeck T, Hellerbrand C, Amann K, Wiesener MS, Höning S, Eckardt KU, Warnecke C (2010). Hypoxia-inducible protein 2 is a novel lipid droplet protein and a specific target gene of hypoxia-inducible factor-1. FASEB J.

[36] Rippo MR, Moretti S, Vescovi S, Tomasetti M, Orecchia S, Amici G, Catalano A, Procopio A (2004). FLIP overexpression inhibits death receptor-induced apoptosis in malignant mesothelial cells. Oncogene.

[37] Dong LF, Swettenham E, Eliasson J, Wang XF, Gold M, Medunic Y, Stantic M, Low P, Prochazka L, Witting PK, Turanek J, Akporiaye ET, Ralph SJ (2007). Vitamin E analogs inhibit angiogenesis by selective apoptosis induction in proliferating endothelial cells: The role of oxidative stress. Cancer Res.

