# Supplementary material for: MicroRNA-126 induces autophagy by altering cell metabolism in malignant mesothelioma
Source: Oncotarget. 2016 Apr 22;7(24):36338–52. doi: 10.18632/oncotarget.8916 (PMC5095004; doi:10.18632/oncotarget.8916)
Supplement: Supplementary file 1 [file oncotarget-07-36338-s001.pdf]

SUPPLEMENTARY FIGURES

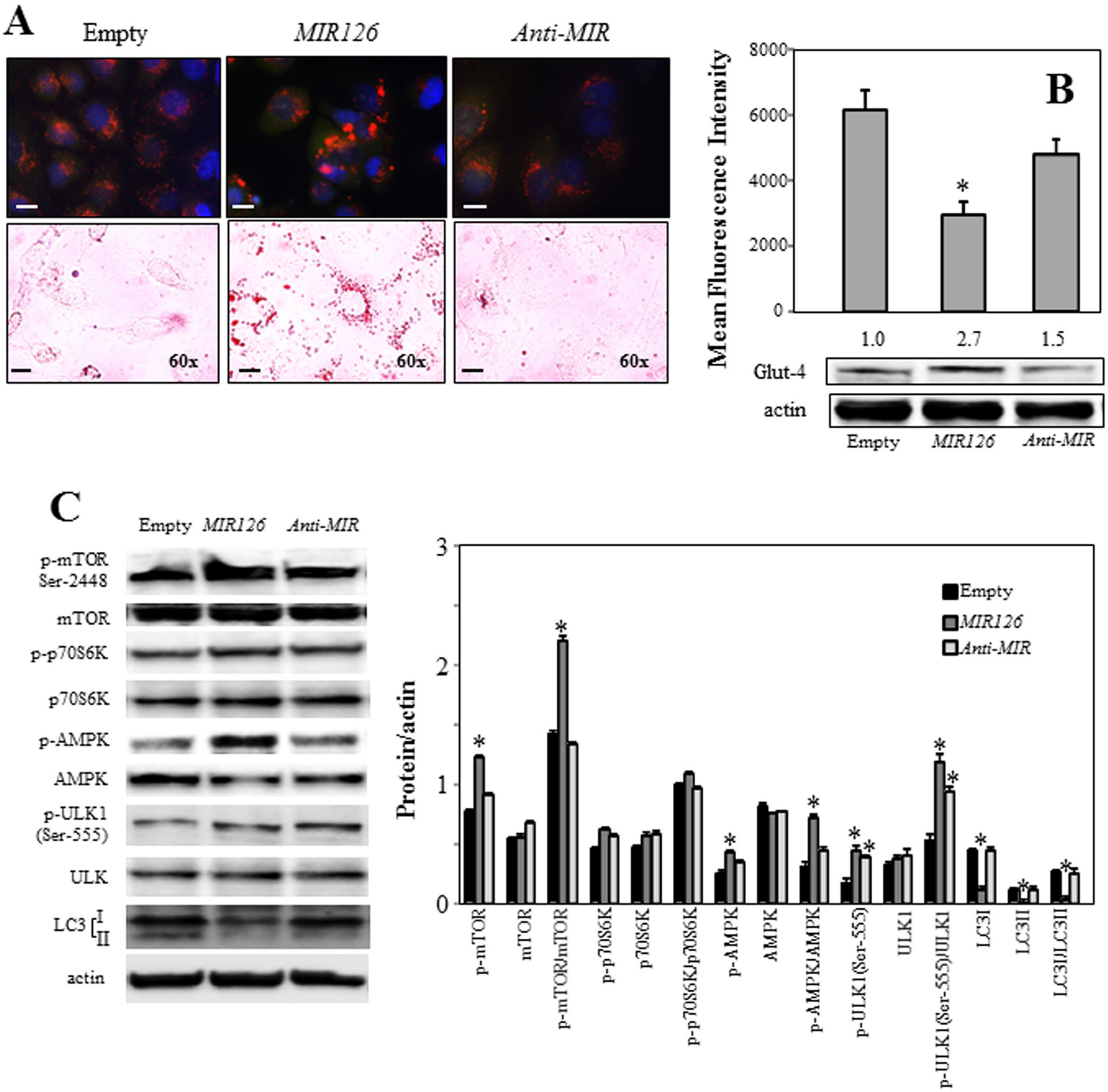

Supplementary Figure S1: H28 cells transfected with empty plasmid-, *MIR126*-, and *MIR126* together with antisense *MIR126* (*anti-MIR*) were evaluated for A. acid vesicle (AV) (acridine orange staining, upper panels), lipid droplet (LD) (Oil Red O staining, lower panels) formation; **B.** Glucose uptake and GLUT-4 expression; **C.** levels of p-mTOR, mTOR and p-p70S6K, p70S6K, p-AMPK, AMPK, p-ULK1 (Ser555), ULK1, LC3I, and LC3II. Densitometric evaluation of the bands is shown relative to actin (right panels). The data shown are mean values ± S.D. derived from five independent experiments. The symbol "*" indicates significant differences compared with empty plasmid-transfected cells.

A

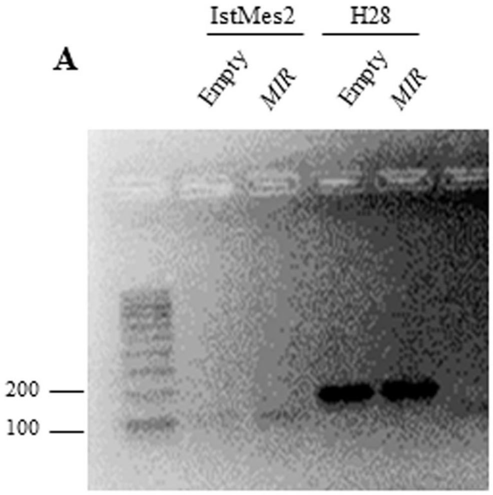

B

MIR126 GAGTAGTCAT
| | | | | | | | | |
IRS1 3' UTR ^{H28} CTCATCAGTAGATGGTACGATGCATCCATTTCAGTTTGTTTACTTTATCCAATCCTCAG
GATTTCATTGACTGAACTGCACGTTCTA

IRS1 3' UTR ^{IstMes2} CTCATCAGTAGATGGTACGA**TGCATCCATTTCAGTTGTTTACT**C**T**G**T**A**CAATCCT**
CAGGAG**TTCATTGACTGAACTGCACGTTCA**

Supplementary Figure S2: IstMes2 cells feature truncated IRS1. IstMes2 lacking the *MIR126*-binding site at the 3'UTR of IRS1 as shown by PCR **A.** and sequencing analysis. **B.** The red sequence represents full *MIR126*-binding site at the 3'UTR of IRS1, which is absent in IstMes2 cells (sequence in grey).

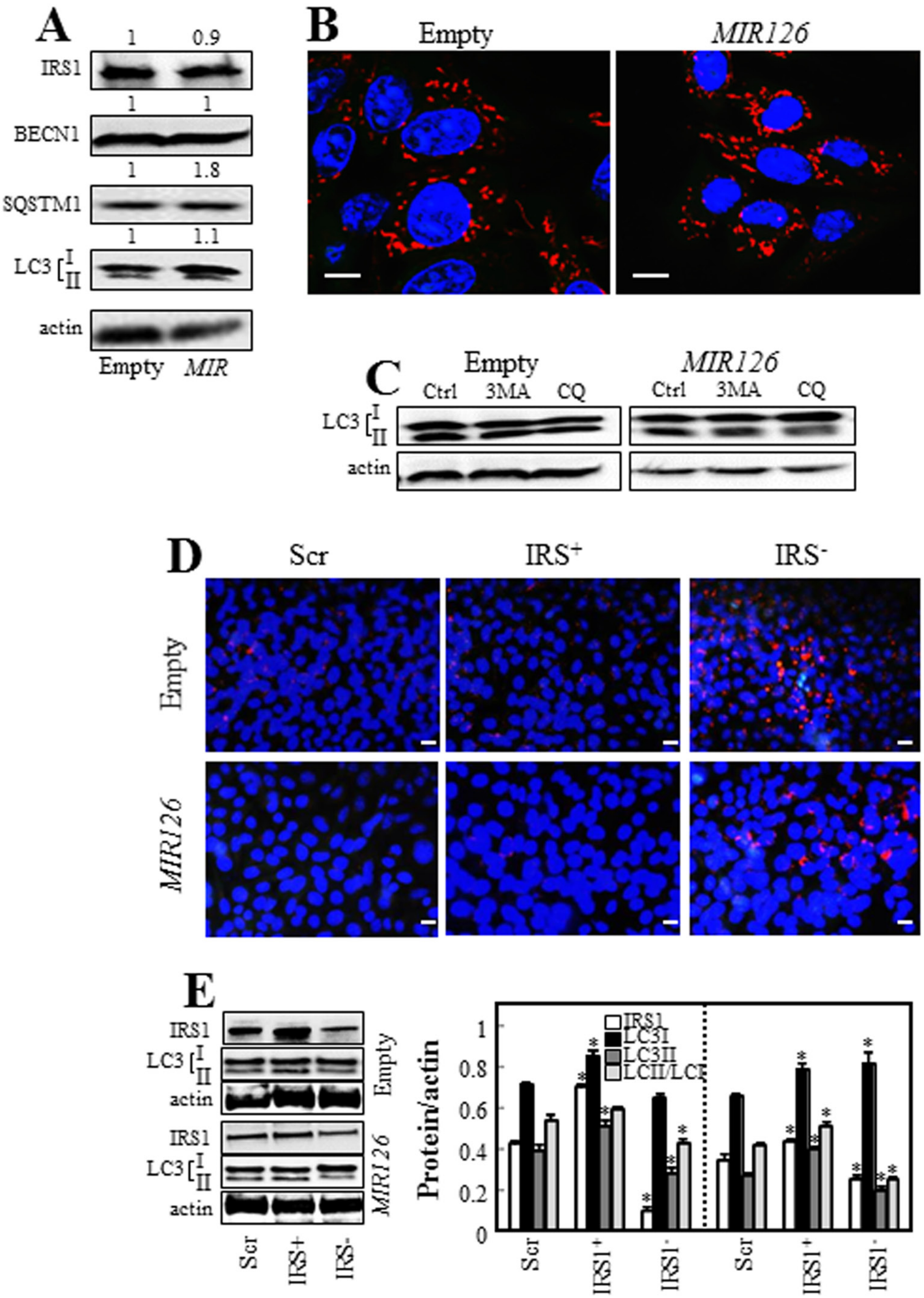

Supplementary Figure S3: Truncated IRS1 inhibits *MIR126*-induced autophagy. Empty plasmid- and *MIR126*-transfected IstMes2 cells with truncated IRS1 lacking the *MIR126*-binding site at the 3'UTR of IRS1 were evaluated for the expression of IRS1, BECN1, SQSTM1 and LC3I/II. Fold changes with respect to parental cells are shown **A.** Autophagy and autophagic flux were evaluated by AO staining **B.** and 3MA or CQ incubation, respectively **C.** Empty plasmid- and *MIR126*-transfected H28 cells and their IRS1-silenced ($IRS1^{-}$) or IRS1-overexpressing ($IRS1^{+}$) counterparts were analyzed for AV formation **D.** and for IRS1 levels and LC3 conversion **E.** Densitometric analysis of IRS1 and LC3 conversion related to actin (right panel). Scale bar for all images = 10 µm. The data shown are mean values ± S.D. derived from three independent experiments. Comparisons among groups were determined by one-way ANOVA with Tukey post-hoc analysis; the symbol "*" indicates significantly different values compared with scramble control with $p < 0.05$.

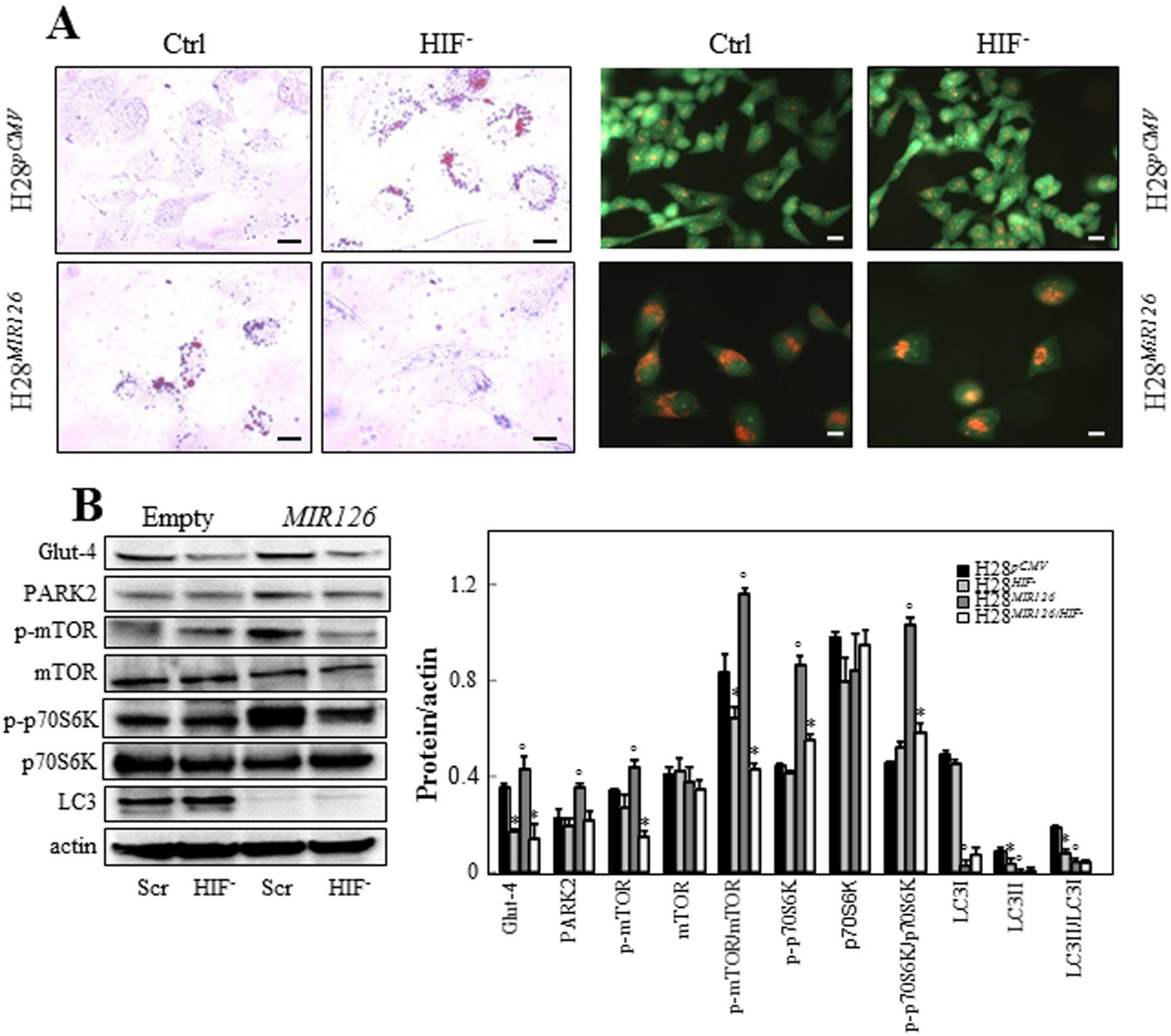

Supplementary Figure S4: HIF1α is involved in *MIR126*-induced lipid droplet formation and mitophagy. A. Empty plasmid- and *MIR126*-transfected H28 cells and their HIF1α-silenced (HIF⁻) counterparts were analyzed for LDs (Oil Red O staining, left panel) and for formation of AVs (orange-red fluorescence, right panel). **B.** Immunoblot of GLUT4, PARK2, p-mTOR, mTOR, p-p70S6K, p70S6K, and LC3. Densitometric analysis of the bands in panel B related to the level of actin is shown in the right panel. Scale bar for all images equals 10 µm. The data shown are mean values ± S.D. derived from three independent experiments. Comparisons among groups were determined by one-way ANOVA with Tukey post-hoc analysis; the symbol "*" indicates significantly different values between empty-plasmid transfected cells and HIF1α-silenced counterparts, and symbol "°" statistically significant difference compared with *MIR126*-transfected cells, with $p < 0.05$.

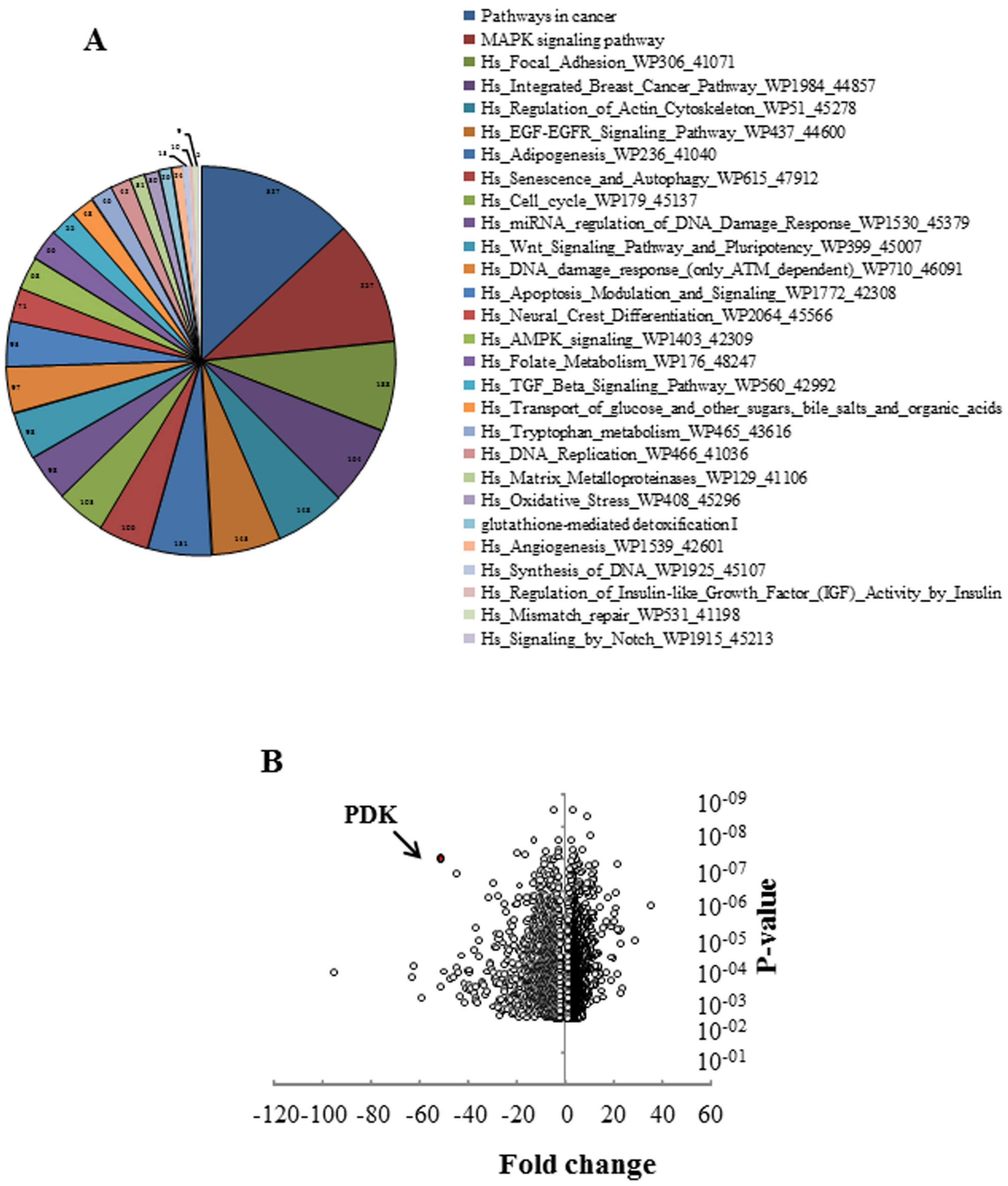

Supplementary Figure S5: Genome-wide gene expression analysis. A. Gene expression analysis was performed in *MIR126*-transfected H28 cells and their empty plasmid-transfected counterparts. Pathway enrichment analysis of *MIR126*-associated molecular signatures derived from H28 cells transfected with *MIR126* and compared with controls. **B.** Up- and downregulated genes in cells overexpressing *MIR126* with respect to empty plasmid-transfected cells were considered differentially expressed if their levels were increased or decreased by more than 2-fold, with $p< 0.05$. Comparisons between samples were determined by unpaired t-test, p-value computation: Asymptotic, Multiple Testing Correction: Benjamini-Hochberg.
